# Do You See What I See? Longitudinal Associations Between Mothers’ and Adolescents’ Perceptions of Their Relationship and Adolescent Internalizing Symptoms

**DOI:** 10.1007/s10802-022-00975-5

**Published:** 2022-09-17

**Authors:** Stefanie A. Nelemans, Stefanos Mastrotheodoros, Leyla Çiftçi, Wim Meeus, Susan Branje

**Affiliations:** 1 Division of Youth and Family, Utrecht University, PO box 80.140, 3508 TC Utrecht, the Netherlands; 2 Utrecht, the Netherlands

**Keywords:** Adolescence, Informant discrepancies, Internalizing symptoms, Parent-adolescent relationship quality, Longitudinal, Latent Congruence Modeling

## Abstract

This 6-year community study examined how discrepancies in mothers’ and adolescents’ perceptions of their relationship were longitudinally associated with adolescent internalizing symptoms, and vice versa. 497 adolescents (57% boys, *M*_age T1_ = 13.03, *SD*_age_ = 0.46) and their mothers reported in 6 annual waves on conflict and warmth in the mother-adolescent relationship and adolescents reported on their depressive and Generalized Anxiety Disorder (GAD) symptoms. Latent Congruence Models suggested that both adolescent depressive and GAD symptoms significantly predicted higher levels of conflict as well as stronger discrepancies in perceptions of conflict 1-year later. In turn, higher levels of conflict significantly predicted both adolescent depressive and GAD symptoms 1-year later. For warmth, lower levels significantly predicted adolescent depressive symptoms 1-year later. Concluding, these findings suggest (1) more systematic evidence for longitudinal associations between conflict than warmth in the mother-adolescent relationship and adolescent internalizing symptoms; (2) support for a *transactional model*, including support for both *interpersonal scar* or *symptom-driven* effects (concerning both levels of and mother-adolescent discrepancies in conflict) and *interpersonal risk* or *relationship-driven* effects (concerning levels of both conflict and warmth); (3) longitudinal effects from adolescent internalizing symptoms to mother-adolescent discrepancies, but not vice versa; and (4) strong consistency in patterns of findings across both adolescent depressive and GAD symptoms, with few differential longitudinal associations with aspects of mother-adolescent relationship quality. Thereby, this study provides a more nuanced understanding of the direction of effects between adolescent internalizing symptoms and both levels of and discrepancies in mothers’ and adolescents’ perceptions of their relationship.

**Supplementary Information:**

The online version contains supplementary material available at 10.1007/s10802-022-00975-5.

## Introduction

Adolescence is a critical period for the development of internalizing problems, such as depressive and Generalized Anxiety Disorder (GAD) symptoms. Not only are these symptoms among the most prevalent forms of psychopathology during adolescence and characterized by strong persistence over time (Kessler et al., 2012), they also substantially impact adolescent functioning and development (Merikangas et al., 2010). Therefore, research that focusses on identifying factors that affect, and are affected by, adolescent internalizing symptoms is essential. In this regard, several aspects of the parent-adolescent relationship have been modestly but systematically associated with adolescent internalizing symptoms (for meta-analyses, see Pinquart, 2017; Yap et al., 2014). Findings generally suggest that higher *levels* of parent-adolescent relationship quality are associated with lower adolescent internalizing symptoms across reporters and methods. Importantly, however, adolescents and parents often perceive their relationship quite differently (Korelitz & Garber, 2016), resulting in so-called *parent-adolescent discrepancies* (a specific form of *informant discrepancies*; De Los Reyes & Ohannessian, 2016). As these discrepancies might reflect maladaptive family processes, such as misunderstanding or lack of awareness in the parent-adolescent relationship, they may be particularly important to consider as a potential risk for adolescent internalizing symptoms (Human et al., 2016). Moreover, both *levels* of parent-adolescent relationship quality (e.g., Nelemans et al., 2014) and parent-adolescent *discrepancies* in relationship quality (Richters, 1992) may not only affect but also *be affected by* adolescent internalizing symptoms. Therefore, the present study aimed to examine longitudinal bidirectional associations (i.e., *direction of effects*) across adolescence among adolescent depressive and GAD symptoms and mother-adolescent relationship quality in terms of levels as well as discrepancies in adolescents’ and mothers’ perceptions.

### Mother-adolescent Relationship Quality and Adolescent Internalizing Symptoms

Even though adolescent depressive symptoms and GAD symptoms are strongly associated (e.g., Brady & Kendall, 1992; Cummings et al., 2014), they represent distinct forms of psychopathology (e.g., Caspi et al., 2014; Hale et al., 2009). Moreover, despite strong associations between these two psychopathological symptoms, meta-analytic evidence suggests they are differentially associated with aspects of the parent-adolescent relationship (e.g., McLeod et al., 2007a, b). That is, parental conflict appears to be more strongly associated with child and adolescent depressive symptoms than anxiety symptoms, and parental warmth appears to be associated with child and adolescent depressive symptoms but less so with anxiety symptoms. These differential associations may reflect the stronger importance of parental acceptance-rejection in youth depression: Low warmth and high conflict may undermine youths’ emotion regulation and the negative parent–child interactions characterized by low acceptance or high rejection may be internalized by youth in negative self-evaluations or a negative view of the world and interpersonal relationships, or negative schemas, thereby increasing vulnerability to particularly depression (e.g., Bolton et al., 2009) and less so anxiety. Because of these potential differential associations, it thus appears to be important to distinguish between adolescent depressive symptoms and GAD symptoms as distinct forms of internalizing symptoms.

Yet, the aforementioned meta-analytical findings do not inform on the *direction of effects* between parent-adolescent relationship quality and adolescent internalizing symptoms. From a developmental psychopathological view (e.g., Rudolph et al., 2016), two critical questions are important to address in this respect: On the one hand, do lower quality mother-adolescent relationships heighten the risk for adolescent internalizing symptoms over time, and, on the other hand, do adolescent internalizing symptoms disturb mother-adolescent relationships over time? With respect to lower mother-adolescent relationship quality potentially increasing risk for adolescent internalizing symptoms over time, interpersonal theories of developmental psychopathology (e.g., Bowlby, 1969; for a review, see Rudolph et al., 2016), and adolescent internalizing symptoms specifically (e.g., Bowlby, 1973, 1980), assume that parent–child relationships constitute a powerful socialization context that may affect adolescent adjustment. In these *interpersonal risk* or *relationship-driven* models, such effects are often referred to as *parent effects*. For example, lower mother-adolescent relationship quality characterized by higher conflict or lower warmth and support may impose interpersonal stress and pose a risk for adolescent internalizing, particularly depressive, symptom development. Similarly, in line with *Attachment Theory*, unpredictable or rejecting parenting (leading to insecure attachment) may provide the foundation for maladaptive internal working models of the self and others and undermine effective emotion and stress regulation, coping, and interpersonal competence, thereby increasing the risk for adolescent internalizing symptom development (for a review, see Rudolph et al., 2016). Supporting these theories, longitudinal evidence suggests that lower mother-adolescent relationship quality is associated with higher adolescent internalizing symptoms over time (e.g., Branje et al., 2010; βs = -0.04 – -0.08; for a meta-analysis, see Yap et al., 2014, *r*s = -0.20 – -0.22).

With respect to adolescent internalizing symptoms potentially disturbing the mother-adolescent relationship over time, interpersonal theories (e.g., *Interactional Theory of Depression*; Coyne, 1976; Joiner & Coyne, 1999) emphasize that adolescent internalizing symptoms may also put a strain on the mother-adolescent relationship. In these *interpersonal scar* or *symptom-driven* models, such effects are often referred to as *child effects*. For example, adolescents with higher internalizing, particularly depressive, symptoms are assumed to rely on negative interpersonal interaction styles, including excessive reassurance seeking and failure to accept support from others, that eventually induce a negative mood in their mothers, which may in turn elicit or reinforce maternal rejection and erode the mother-adolescent relationship. Similarly, the *Stress Generation Perspective* (Hammen, 2006) posits that adolescent depressive symptoms generate stress in interpersonal relationships, which may in turn deteriorate the quality of the mother-adolescent relationship. These child effects may be especially likely to occur in adolescence, as major developmental changes in the parent–adolescent relationship take place and this relationship becomes more horizontal, interdependent, and symmetrical (Laursen & Collins, 2009). Supporting these theories, longitudinal evidence suggests that higher adolescent internalizing symptoms are associated with lower mother-adolescent relationship quality over time (e.g., Branje et al., 2010, βs = -0.08 – -0.12; Nelemans et al., 2014, βs = -0.09 – -0.13). Taken together, theories and empirical findings support a *transactional perspective* (Rudolph, 2009; Sameroff, 2009), in which lower mother-adolescent relationship quality may predict higher adolescent internalizing symptoms over time as well as vice versa.

### Mother-adolescent Discrepancies and Adolescent Internalizing Symptoms

It is important to acknowledge that adolescents and mothers may perceive the quality of their relationship quite differently (De Los Reyes & Ohannessian, 2016) and these *mother-adolescent discrepancies* may substantially impact internalizing symptoms. Overall, adolescents’ and parents’ reports of family functioning, including mothers’ and adolescents’ reports of their relationship quality (Pelton & Forehand, 2001), show low-to-moderate correspondence (Korelitz & Garber, 2016). For example, due to their larger investment in the family, mothers may perceive the mother-adolescent relationship more positively and optimistically than their adolescents, causing mother-adolescent discrepancies (i.e., *Generational Stake Hypothesis*; Bengtson & Kuypers, 1971; for empirical support in adolescence, see e.g., the meta-analysis by Korelitz & Garber, 2016). Importantly, however, there is substantial variation between families in patterns of convergence and divergence between reports of adolescents and parents. This variation in parent-adolescent discrepancies, including discrepancies in mothers’ and adolescents’ perceptions of their relationship quality, is now widely acknowledged to yield valid and meaningful information (De Los Reyes, 2011, 2013) and research has increasingly sought to explain or improve interpretability of parent-adolescent discrepancies in several areas (e.g., youth mental health; De Los Reyes, 2013; De Los Reyes & Kazdin, 2005), including family functioning (De Los Reyes & Ohannessian, 2016). The Operations Triad Model (De Los Reyes et al., 2013), which has recently been modified for use in research on family functioning (De Los Reyes & Ohannessian, 2016), is among the leading frameworks to guide multi-informant research. Central to this model is that the meaning of patterns of convergence and divergence between reports of adolescents and parents should be understood in association with independent criterion measures, such as adolescent psychopathological symptoms. In line with *interpersonal risk* or *relationship-driven* models, parent-adolescent discrepancies are both theoretically and empirically considered as potential risk for (later) youth psychopathological symptoms, whereas in line with *interpersonal scar* or *symptom-driven* models, youth psychopathological symptoms may also be considered as potential risk for (later) parent-adolescent discrepancies.

One example of an interpersonal risk model are *Goodness of Fit* models (e.g., Lerner et al., 1986; Thomas & Chess, 1977), which posit that mother-adolescent discrepancies in how they perceive their relationship reflect a form of *misfit* that increases the risk for adolescent internalizing symptom development. Such discrepancies or misfit may result from a mismatch between parental demands (in the form of attitudes, values, or expectations) and youths’ behavior, which may be particularly likely to occur in adolescence, because several new psychological and social/contextual demands need to be negotiated and major developmental changes (e.g., realignment) in the parent–adolescent relationship take place (Laursen & Collins, 2009). Together with adolescents’ strife for separation, individuation, and increased autonomy from early adolescence onwards, adolescence thus appears to be a particularly important developmental period to study mother-adolescent discrepancies as potential risk for adolescent internalizing symptom development during an already critical development period for these symptoms (Kessler et al., 2012). Similarly, *Stage-Environment Fit Theory* (Eccles et al., 1993) posits that adolescence is characterized by a *developmental mismatch* between the needs of developing adolescents and the opportunities provided by their social environments, including their parents. This may result in increased mother-adolescent discrepancies that, in turn, increase the risk for adolescent internalizing symptom development. Supporting these theories and in line with the Diverging Operations in the Operations Triad Model (De Los Reyes & Ohannessian, 2016), empirical evidence from longitudinal studies suggests that larger discrepancies in mother-adolescent relationship quality are associated with higher adolescent internalizing symptoms over time (e.g., Guion et al., 2009; Hou et al., 2018; Pelton & Forehand, 2001). However, in line with Converging Operations in the Operations Triad Model (De Los Reyes & Ohannessian, 2016), it should also be acknowledged that *convergence* in mother-adolescent reports of *risk factors* in their relationship has been associated with increased adolescent internalizing symptoms over time (e.g., Nelemans et al., 2016), whereas convergence in mother-adolescent reports of *protective factors* in their relationship has been associated with decreased adolescent internalizing symptoms over time (e.g., Laird & De Los Reyes, 2013).

Alternatively, according to the *Depression-Distortion Hypothesis,* adolescent internalizing symptoms have been suggested to predict stronger mother-adolescent discrepancies in perceptions of their relationship over time, as cognitive biases or biased negative experiences of social relationship may cause a more negative adolescent perception of the mother-adolescent relationship and thereby stronger mother-adolescent discrepancies (Richters, 1992). Little is known empirically on whether youth psychopathological symptoms may also predict stronger parent-adolescent discrepancies over time, in line with *interpersonal scar* or *symptom-driven* models. Some indications from cross-sectional studies (Chi & Hinshaw, 2002; De Los Reyes et al., 2008) suggest that (maternal) internalizing symptoms may indeed be associated with stronger mother-adolescent discrepancies. Clearly, more longitudinal research is needed that considers potential *bidirectional* longitudinal associations between mother-adolescent discrepancies in perceptions of their relationship quality and adolescent internalizing symptoms.

### The Present Study

The current study examined potential bidirectional longitudinal associations across adolescence among adolescent internalizing symptoms and both levels of and discrepancies in adolescents’ and mothers’ perceptions of mother-adolescent relationship quality. We specifically examined potential differential longitudinal associations among adolescent depressive and GAD symptoms and both conflict and warmth in the mother-adolescent relationship (as reflections of negative and positive aspects of mother-adolescent relationship quality, respectively) to explore robustness of findings and get to a more nuanced understanding of direction of effects in these associations over time.

In line with interpersonal risk models (for a review, see Rudolph et al., 2016), we hypothesized that lower mother-adolescent relationship quality (i.e., higher conflict and lower warmth) was associated with higher adolescent internalizing symptoms over time. In addition, in line with interpersonal scar or symptom-driven models (Coyne, 1976; Hammen, 2006; Joiner & Coyne, 1999), we hypothesized that higher adolescent internalizing symptoms were associated with lower mother-adolescent relationship quality over time. Furthermore, in line with Goodness of Fit models (Lerner et al., 1986; Thomas & Chess, 1977), Stage-Environment Fit Theory (Eccles et al., 1993), and the Diverging Operations in the Operations Triad Model (De Los Reyes & Ohannessian, 2016), we hypothesized that larger discrepancies in mother-adolescent relationship quality were associated with higher adolescent internalizing symptoms over time. We explored whether higher adolescent internalizing symptoms were associated with larger discrepancies in mother-adolescent relationship quality over time, in line with the Depression-Distortion Hypothesis (Richters, 1992). In sum, we thus hypothesized bidirectional longitudinal associations among adolescent depressive and GAD symptoms and both levels of and discrepancies in adolescents’ and mothers’ perceptions of warmth and conflict in their relationship, in line with transactional models (Rudolph, 2009; Sameroff, 2009). Because of robust gender differences in adolescent internalizing problems (McLean & Anderson, 2009; Rudolph, 2009), we conducted sensitivity analyses including adolescent gender as a time-invariant covariate.

Whereas much of the past research in the field has relied on methodologically limited methods for measuring adolescent–parent discrepancies, such as difference scores (for a review, see Laird & De Los Reyes, 2013), and used either cross-sectional designs or longitudinal designs in which typically only one direction of effects (typically the effect of parent-adolescent discrepancies on adolescent functioning) has been examined, we applied Latent Congruence Modeling (LCM; Cheung, 2009) as a more adequate alternative to the analysis of informant discrepancies. Importantly, LCM allows to examine potential bidirectional longitudinal associations between adolescent internalizing symptoms and mother-adolescent relationship quality above and beyond potential cross-sectional associations and stability in the constructs over time. Furthermore, LCM considers both levels and discrepancies in adolescents’ and mothers’ perceptions of mother-adolescent relationship quality in the same analytical model and thereby captures unique associations with adolescent internalizing symptoms. Finally, LCM includes the critical step of testing for *measurement invariance* in perceptions of the mother-adolescent relationship across informants (as well as across time), to ensure that the mother-adolescent relationship is similarly assessed across informants and time and valid conclusions on patterns of convergence and discrepancy can be made concerning reports of adolescents and mothers of their relationship across time. Thereby, this study extends prior research in several ways and its findings add to the current literature in important ways.

## Method

### Study Design and Procedure

Data were part of the ongoing longitudinal community study Research on Adolescent Development And Relationships (RADAR-Young; http://doi.org/10.17026/dans-zrb-v5wp). Participants were recruited from randomly selected primary schools in the western and central regions of the Netherlands. From these schools, adolescents in the final grade were screened for (sub)clinical levels of externalizing symptoms and subsequently adolescents from two-parent families were randomly selected for participation, with about half of the sample being selected from the group of adolescents with (sub)clinical levels of externalizing symptoms. Before the start of the study in 2006, adolescents and their parents received a description of the study and provided active written informed consent to participate. Data collection started in the new academic year, when all participants attended the first year of secondary school. The final sample thereby consisted of only one adolescent (with some exceptions including two adolescents) from every secondary school. Adolescents and their parents were invited to complete annual questionnaires during a home visit across six annual measurement waves and received a small monetary compensation for participation. This study was approved by the board of the local research institute and the Faculty Ethical Committee of Utrecht University, the Netherlands.

### Participants

Participants in this ongoing longitudinal community study were 497 adolescents (57% boys, *M*_age_ T_1_ = 13.03 years, *SD*_age_ = 0.46) of mainly Dutch-Caucasian ethnicity (95%) and their mothers (*M*_age_ T_1_ = 44.41 years, *SD*_age_ = 4.45). Most adolescents indicated that they lived in two-parent families (85.2%) at the start of the study and 10.8% of the families was characterized by low SES (based on parents’ reported job level).

Sample attrition was low across the six yearly measurement waves included in the current study, with 426 of the 497 adolescents (85.7%) and 420 of the 497 mothers (84.5%) still participating at the sixth measurement wave. Adolescents still participating at the last measurement wave were slightly younger than those dropping out, *F*(1, 495) = 5.90, *p* = 0.02, partial η^2^ = 0.01, but they did not differ with respect to gender, χ^2^(1) = 0.79, *p* = *0.*38, or the variables of interest (i.e., depressive symptoms, GAD symptoms, and adolescent-reports of conflict and warmth in the mother-adolescent relationship) at the start of the study, *F*(4, 480) = 1.37, *p* = 0.25. Mothers still participating at the last measurement wave were slightly older than those dropping out, *F*(1, 495) = 4.75, *p* = 0.03, partial η^2^ = 0.01, but they did not differ with respect to the variables of interest (i.e., mother-reports of conflict and warmth in the mother-adolescent relationship) at the start of the study, *F*(2, 491) = 0.26, *p* = 0.77.

### Measures

#### Adolescent Depressive Symptoms

The shortened Dutch version of the Reynolds Adolescent Depression Scale – second edition (RADS-2; Reynolds, 2000) was used to assess adolescent depressive symptoms. The shortened RADS-2 is a 23-item self-report questionnaire (excluding the 7-item anhedonia subscale from the full RADS-2 version) that was completed by adolescents. Items were measured on a 4-point scale ranging from 1 (*almost never*) to 4 (*usually*). A sample item reads “I am sad”. In this study, higher scores reflected higher mean levels of adolescent depressive symptoms, with a potential range of 1 to 4. Internal consistency was good in our sample across all waves, with Cronbach’s alpha ranging from 0.93–0.95. The RADS-2 has proven to be internally consistent in samples of adolescents with alphas ranging from 0.93 to 0.95 in a non-clinical setting and 0.92 to 0.98 in a clinical setting and has shown measurement invariance across gender and age. In addition, the RADS-2 has proven to differentiate between subgroups of adolescents with mood disorders versus other psychiatric disorders with medium accuracy and RADS-2 scores have shown strong correlations with suicide ideation and behavior and moderate correlations with hopelessness, adaptation, and self-concept (Fonseca-Pedrero et al., 2010; Osman et al., 2010).

#### Adolescent GAD Symptoms

The 9-item GAD subscale of the Dutch version of the Screen for Child Anxiety Related Emotional Disorders (SCARED; Birmaher et al., 1997) was used to assess adolescent GAD symptoms. The SCARED is a self-report questionnaire that was completed by adolescents. Items were rated on a 3-point scale, ranging from 1 (*almost never*) to 3 (*often*). A sample reads “I am a worrier”. In this study, higher scores reflected higher mean levels of adolescent GAD symptoms, with a potential range of 1 to 3. Internal consistency for the GAD subscale was good in our sample across waves, with Cronbach’s alpha ranging between 0.85–0.91. The SCARED has shown a robust five-factor structure congruent with its theoretical conceptualization (Birmaher et al., 1997; Crocetti et al., 2009) and has proven to be internally consistent in samples of adolescents with a mean alpha in a meta-analysis of 0.81 [95% CI = 0.78–0.83] for the GAD subscale across clinical and non-clinical settings across adolescence (Hale et al., 2011). In addition, the SCARED has proven to differentiate between adolescents with anxiety disorders versus nonanxiety psychiatric disorders as well as adolescents with GAD versus other anxiety disorders (Birmaher et al., 1997).

#### Mother-adolescent Relationship Quality

The 6-item conflict subscale and the shortened 5-item^1^ warmth subscale of the Dutch version of the Network of Relationships Inventory (NRI; Furman & Buhrmester, 1985) was used to assess both negative and positive aspects of the mother-adolescent relationship as perceived by both adolescents and mothers. The NRI is a self-report questionnaire that was completed by both adolescents and their mothers to capture their perception of the mother-adolescent relationship. Items were rated on a 5-point scale, ranging from 1 (*little or none*) to 5 (*the most*). Sample items read “How much do you and your child/mother argue with each other?” for conflict and “How sure are you that this relationship will last no matter what?” for warmth perceived by the mother and the adolescent, respectively. In this study, higher scores reflected higher mean levels of conflict or warmth in the mother-adolescent relationship, with a potential range of 1 to 5.

Internal consistency for both subscales was good in our sample across informants and across waves, with Cronbach’s alpha ranging from 0.90–0.95 for adolescent reports on conflict, 0.80–0.88 for adolescent reports on warmth, 0.90–0.92 for mother reports on conflict, and 0.74–0.80 for mother reports on warmth. The NRI has proven to be internally consistent in samples of adolescents with alphas of 0.93 and 0.94 for conflict and warmth in the mother-adolescent relationship, respectively, and scores have been correlated with coders’ ratings of communication, conflict, and dyadic positively in observed mother-adolescent interactions (Furman & Buhrmester, 2009).

### Statistical Analyses

#### Missing Data

Most missing data on the variables of interest were the result of dropout, whereas some missing data were due to unavailability of participants at a specific measurement wave or participants’ decision not to complete certain parts of the questionnaire at a specific measurement wave. Little’s Missing Completely at Random (MCAR) test showed a normed χ^2^ (χ^2^/df) of 1.09, χ^2^(5484) = 5976.71, suggesting that the data were likely missing at random (Bollen, 1989). Missing data were handled in M*plus* with Full Information Maximum Likelihood (Muthén & Muthén, 2017).

#### Measurement Invariance Analyses

As a preliminary step required before conducting the Latent Congruence Models (LCMs; Cheung, 2009), Confirmatory Factor Analyses (CFAs) concerning longitudinal measurement invariance and measurement invariance across informants were conducted. Thus, for each construct the configural invariance (baseline) model was compared with the metric invariance model, in which factor loadings were constrained to be equal across time/informants, the scalar invariance model with equal item intercepts across time/informants, and the strict invariance model with equal residual variances across time/informants. Model comparisons were conducted considering changes in fit indices (Chen, 2007). Findings indicated strict measurement invariance for all constructs, both longitudinally and across informants (for details, please see Online Resource 1). This suggests that conflict and warmth in the mother-adolescent relationship were assessed similarly across adolescence for both mothers and adolescents (i.e., longitudinal measurement invariance), as well as across informants (i.e., longitudinal measurement invariance across mothers and adolescents).

#### Main Analyses

After longitudinal measurement invariance was established across informants, four LCMs (Cheung, 2009) were constructed to examine longitudinal associations among mother-adolescent discrepancies in reports of conflict and warmth and adolescent depressive and GAD symptoms, respectively. LCM uses a structural equation modeling (SEM) framework to capture discrepancies between informants within two higher-order latent variables that build on the aforementioned CFAs (run to test longitudinal measurement invariance across informants): One latent variable captures the mean-level of conflict/warmth in the mother-adolescent relationship as reported by both informants, and the other latent variable captures the discrepancy between informants on their reports of conflict/warmth in the mother-adolescent relationship (see Fig. 2.1 in Online Resource 2 for a simplified graphical representation of this part of the LCMs). In our parameterization of the LCM, larger discrepancies represented higher levels of conflict/warmth reported by adolescents compared to their mothers. So essentially, the difference in reports of mothers and adolescents on conflict/warmth in their relationship at each time point is captured within a latent variable. Within this SEM framework, researchers can examine both antecedents and consequences of informant discrepancies over time, above and beyond concurrent associations and temporal stability in constructs over time. LCM hereby overcomes limitations associated with the use of difference scores, for example concerning reliability and validity (Edwards, 1994, 2002; Laird & De Los Reyes, 2013) and polynomial regression analysis, for example concerning the inclusion of both antecedents and consequences of informant discrepancies (Cheung, 2009).

Analyses were conducted separately for conflict and warmth in the mother-adolescent relationship and adolescent depressive symptoms and GAD symptoms, resulting in four bivariate LCMs. The baseline LCMs included all concurrent (i.e., within-time) associations between the latent mean-level and discrepancy factors of conflict or warmth and adolescent depressive or GAD symptoms, 1-year autoregressive paths within constructs over time, and all possible 1-year cross-lagged associations between the latent mean-level and discrepancy factors of conflict or warmth and adolescent depressive or GAD symptoms. For reasons of parsimony–and because there were no specific hypotheses regarding non-stationarity of the associations–, all longitudinal parameters were constrained to be time invariant in the baseline LCMs. Specifically, by constraining similar parameters, to be equal over time, the analysis only provides one estimate for each parameter that applies across all waves. For example, by constraining the cross-lagged effect of level of conflict in the mother-adolescent relationship at wave_x_ on later adolescent depressive symptoms at wave_x + 1_ to be equal over time, the cross-lagged effect of level of conflict at wave 1 on depressive symptoms at wave 2 is equal to the cross-lagged effect of level of conflict at wave 2 on depressive symptoms at wave 3, and so on. To examine potential changes in the concurrent, autoregressive, and cross-lagged associations across time, we tested whether freeing each specific set of longitudinal parameters in a stepwise manner resulted in a significantly better model fit. The comparative fit of models was tested using Satorra-Bentler scaled chi-square difference tests (Δχ^2^_SB_; Satorra & Bentler, 2001). In addition, sensitivity analyses included adolescent gender as a time-invariant covariate of all variables in the final LCMs.

## Results

### Descriptive Statistics

Table S3.1 in Online Resource 3 provides an overview of the means and standard deviations of all study variables as well as descriptive tests of congruency between mother- and adolescent-reports of conflict and warmth by paired *t*-tests for mean-level differences and by bivariate correlations. Adolescents reported slightly higher levels of both conflict and warmth than their mothers across adolescence (*p*s < 0.001). Also, adolescent-reports correlated significantly moderately to strongly with mother-reports across adolescence, *r*s = 0.45 – 0.54 for conflict and *r*s = 0.21 – 0.42 for warmth, respectively.

Table S3.2 in Online Resource 3 presents a summary of concurrent (i.e., at the same moment in time) associations between reports of adolescents and mothers on conflict and warmth and adolescent depressive and GAD symptoms across time.^2^ Correlations suggested significant, moderately strong concurrent associations between adolescent reports of conflict (*r*s = 0.29 – 0.37 across waves) and warmth (*r*s = -0.21 – -0.31 across waves) with adolescent depressive symptoms. In addition, significant moderately strong concurrent associations between adolescent reports of conflict and adolescent GAD symptoms were found (*r*s = 0.19 – 0.27 across waves). Adolescent reports of warmth were not always significantly associated with adolescent GAD symptoms, and significant concurrent associations were weak (*r*s = -0.06 – -0.16 across waves). Furthermore, comparable but less strong concurrent associations were found between mother reports of conflict and warmth with adolescent depressive (*r*s = 0.23 – 0.30 and *r*s = -0.09 – -0.16 across waves, respectively) and GAD symptoms (*r*s = 0.11 – 0.20 and *r*s = -0.09 – 0.02 across waves, respectively).

### Mother-adolescent Discrepancies and Adolescent Internalizing Symptoms

#### Conflict

**Fig. 1:**
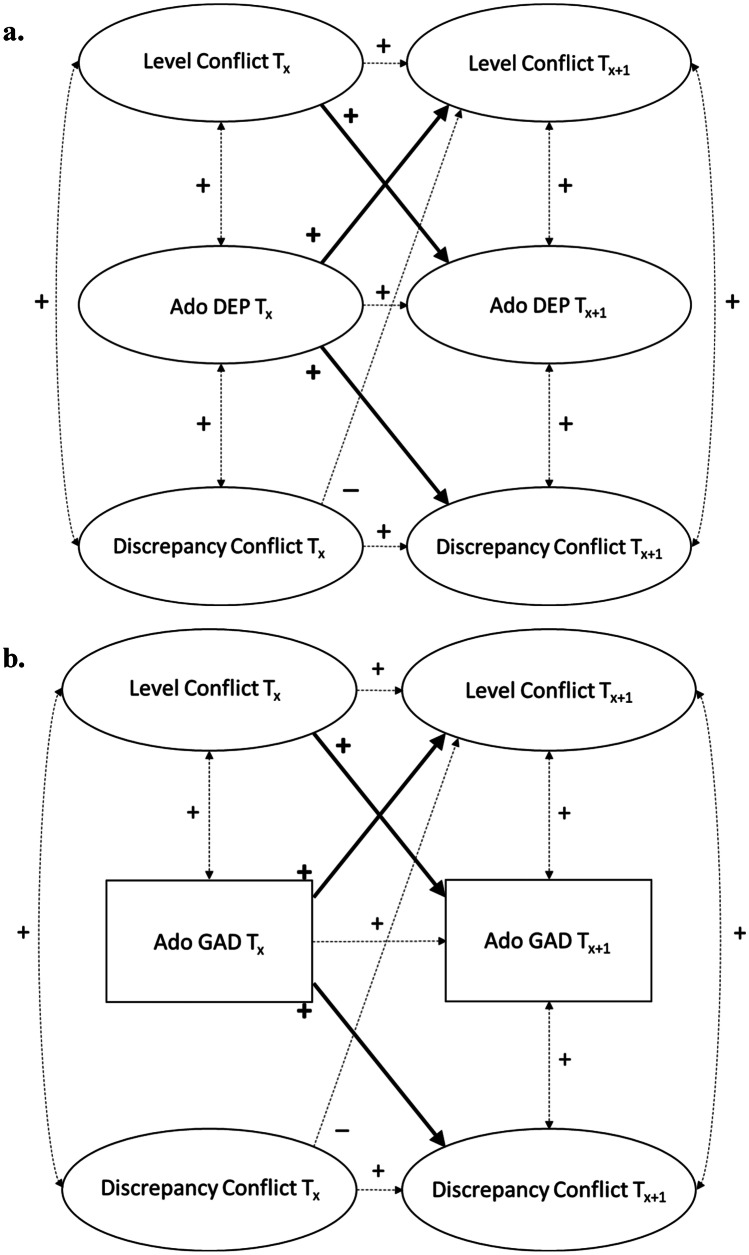
Simplified Visualization of the Main Findings of the Latent Congruence Models on the Association between Conflict in the Mother-Adolescent Relationship and **(a)** Adolescent Depressive Symptoms / **(b)** Adolescent GAD Symptoms (*N* = 497). *Note.* Only significant associations are visualized. The significant main findings are presented in bold lines, whereas the other significant associations are presented in dashed lines. *Ado* adolescent, *DEP* depressive symptoms, *GAD* Generalized Anxiety Disorder symptoms

The time-invariant baseline LCM showed acceptable to good fit to the data for the association between conflict in the mother-adolescent relationship and both adolescent depressive symptoms, χ^2^_SB_(3086) = 5170.51, CFI = 0.920, RMSEA [90% CI] = 0.037 [0.035, 0.039], SRMR = 0.085, and adolescent GAD symptoms, χ^2^_SB_(3086) = 5233.99, CFI = 0.918, RMSEA [90% CI] = 0.037 [0.036, 0.039], SRMR = 0.084. Stepwise freeing all bivariate within-time correlated changes, Δχ^2^_SB_(4) ≤ 4.09, *p*s ≥ 0.394 and Δχ^2^_SB_(4) ≤ 2.28, *p*s ≥ 0.684, and cross-lagged paths, Δχ^2^_SB_(4) ≤ 5.38, *p*s ≥ 0.250 and Δχ^2^_SB_(4) ≤ 7.08, *p*s ≥ 0.132, over time did not significantly improve model fit in neither the adolescent depressive nor GAD symptom model, respectively. For reasons of parsimony, all longitudinal parameters were therefore kept constrained to be equal over time in both models. Results of the final LCMs are reported in Table 1 and a summary of the main findings is visualized in Fig. 1.

**Table 1:** Overview of Results of the Latent Congruence Models (*N* = 497)

Model / parameter	Conflict		Warmth	
	*b* (*SE*)	*r* / β	*b* (*SE*)	*r* / β
Adolescent depressive symptoms				
** Ado DEP**_**Tx**_ **→** **Level RQ**_**Tx+1**_	0.039 (0.016)^*^	**0.039 – 0.043**	**-0.012 (0.014)**	**-0.013 – -0.015**
** Ado DEP**_**Tx**_ **→ Discrepancy RQ**_**Tx+1**_	0.062 (0.023)^**^	**0.054 – 0.061**	**-0.027 (0.021)**	**-0.022 – -0.025**
**Level RQ**_**Tx**_ **→ Ado DEP**_**Tx+1**_	0.076 (0.023)^***^	**0.063 – 0.076**	-0.058 (0.024)^*^	**-0.037 – -0.051**
**Discrepancy RQ**_**Tx**_ **→ Ado DEP**_**Tx+1**_	**-0.009 (0.017)**	**-0.009 – -0.010**	**-0.019 (0.017)**	**-0.019 – -0.022**
Level RQ_Tx_ → Discrepancy RQ_Tx+1_	-0.047 (0.030)	-0.038 – -0.044	-0.081 (0.033)^*^	-0.048 – -0.065
Discrepancy RQ_Tx_ → Level RQ_Tx+1_	-0.058 (0.019)^**^	-0.062 – -0.066	-0.065 (0.016)^***^	-0.079 – -0.088
T_1_ correlation Discrepancy RQ – Ado DEP	0.031 (0.014)^*^	0.119	-0.042 (0.016)^**^	-0.161
T_1_ correlation Level RQ – Ado DEP	0.081 (0.015)^***^	0.366	-0.045 (0.010)^***^	-0.264
T_1_ correlation Discrepancy RQ – Level RQ	0.034 (0.017)^*^	0.141	0.009 (0.015)	0.048
T_2_-T_6_ correlations Discrepancy RQ – Ado DEP	0.021 (0.005)^***^	0.108 – 0.144	-0.028 (0.009)^***^ – 0.007 (0.008)^a^	-0.19 – 0.056
T_2_-T_6_ correlations Level RQ – Ado DEP	0.027 (0.004)^***^	0.192 – 0.246	-0.013 (0.003)^***^	-0.123 – -0.146
T_2_-T_6_ correlations Discrepancy RQ – Level RQ	0.064 (0.007)^***^	0.399 – 0.470	0.063 (0.008)^***^	0.557 – 0.598
Autoregressive paths Ado DEP	0.690 (0.027)^***^	0.627 – 0.723	0.702 (0.026)^***^	0.634 – 0.738
Autoregressive paths Discrepancy RQ	0.592 (0.032)^***^	0.555 – 0.626	0.713 (0.029)^***^	0.646 – 0.736
Autoregressive paths Level RQ	0.768 (0.025)^***^	0.709 – 0.777	0.869 (0.021)^***^	0.756 – 0.848
Adolescent GAD symptoms				
**Ado GAD**_**Tx**_ **→ Level RQ**_**Tx+1**_	0.045 (0.018)^*^	**0.036 – 0.040**	**-0.004 (0.015)**	**-0.003 – -0.004**
**Ado GAD**_**Tx**_ **→ Discrepancy RQ**_**Tx+1**_	0.076 (0.029)^**^	**0.052 – 0.062**	**-0.021 (0.023)**	**-0.014 – -0.017**
**Level RQ**_**Tx**_ **→ Ado GAD**_**Tx+1**_	0.051 (0.016)^***^	**0.053 – 0.059**	**-0.010 (0.018)**	**-0.008 – -0.011**
**Discrepancy RQ**_**Tx**_ **→ Ado GAD**_**Tx+1**_	**-0.011 (0.014)**	**-0.014 – -0.015**	**-0.011 (0.014)**	**-0.014 – -0.015**
Level RQ_Tx_ → Discrepancy RQ_Tx+1_	-0.039 (0.029)	-0.031 – -0.037	-0.073 (0.032)^*^	-0.043 – -0.058
Discrepancy RQ_Tx_ → Level RQ_Tx+1_	-0.058 (0.019)^**^	-0.063 – -0.066	-0.064 (0.016)^***^	-0.077 – -0.086
T_1_ correlation Discrepancy RQ – Ado GAD	0.020 (0.011)	0.099	-0.010 (0.012)	-0.049
T_1_ correlation level RQ – Ado GAD	0.046 (0.011)^***^	0.264	-0.021 (0.007)^**^	-0.157
T_1_ correlation Discrepancy RQ – Level RQ	0.034 (0.017)^*^	0.141	0.008 (0.015)	0.045
T_2_-T_6_ correlations Discrepancy RQ – Ado GAD	0.015 (0.004)^***^	0.102 – 0.126	-0.002 (0.004)	-0.014 – -0.017
T_2_-T_6_ correlations level RQ – Ado GAD	0.011 (0.003)^***^	0.100 – 0.120	-0.001 (0.002)	-0.010 – -0.011
T_2_-T_6_ correlations Discrepancy RQ – Level RQ	0.064 (0.008)^***^	0.399 – 0.475	0.063 (0.008)^***^	0.546 – 0.600
Autoregressive paths Ado GAD	0.729 (0.025)^***^	0.658 – 0.746	0.740 (0.025)^***^	0.668 – 0.753
Autoregressive paths Discrepancy RQ	0.591 (0.032)^***^	0.555 – 0.625	0.712 (0.029)^***^	0.646 – 0.734
Autoregressive paths Level RQ	0.774 (0.024)^***^	0.711 – 0.785	0.872 (0.021)^***^	0.759 – 0.849

The main findings in relation to the hypotheses are presented in bold

*Ado* adolescent, *DEP* depressive symptoms, *GAD* Generalized Anxiety Disorder symptoms, *RQ* mother-adolescent relationship quality (referring to either conflict or warmth in the mother-adolescent relationship)

*p ≤ 0.050; ** *p* ≤ 0.010; *** *p* ≤ 0.001

^a^In the final LCM, all longitudinal parameters were kept constrained to be equal over time, except for the freely estimated within-time correlated change across time between adolescent depressive symptoms and discrepancies between mothers and adolescents in their reports of warmth. This result refers to the range in correlated change across different waves

Most important to this study, results suggested a similar pattern of findings for adolescent depressive symptoms and adolescent GAD symptoms. Specifically, as hypothesized both depressive and GAD symptoms significantly but weakly (βs = 0.04, *p* = 0.013 and βs = 0.04, *p* = 0.011, respectively) predicted higher levels of conflict 1-year later, in line with interpersonal scar or symptom-driven models. As hypothesized, higher levels of conflict also significantly but weakly predicted higher adolescent depressive (βs = 0.06 – 0.08, *p* = 0.001) and GAD (βs = 0.05 – 0.06, *p* = 0.001) symptoms 1-year later, in line with interpersonal risk models. These results suggest positive bidirectional longitudinal associations between levels of conflict in the mother-adolescent relationship and adolescent internalizing symptoms over time, consistent with our hypotheses in line with transactional models. In addition, both adolescent depressive (βs = 0.05 – 0.06, *p* = 0.008) and GAD (βs = 0.05 – 0.06, *p* = 0.009) symptoms predicted significantly larger discrepancies between mothers and adolescents in their reports of conflict 1-year later (i.e., increased adolescent-reported conflict compared to mother-reported conflict). Yet, in contrast to our hypothesis, discrepancies in reports of conflict did not significantly predict adolescent depressive (*p* = 0.60) or GAD (*p* = 0.41) symptoms 1-year later. These results suggest a positive unidirectional longitudinal association from adolescent internalizing symptoms to discrepancies in reports of conflict, in line with the Depression-Distortion hypothesis (Richters, 1992).

#### Warmth

**Fig. 2:**
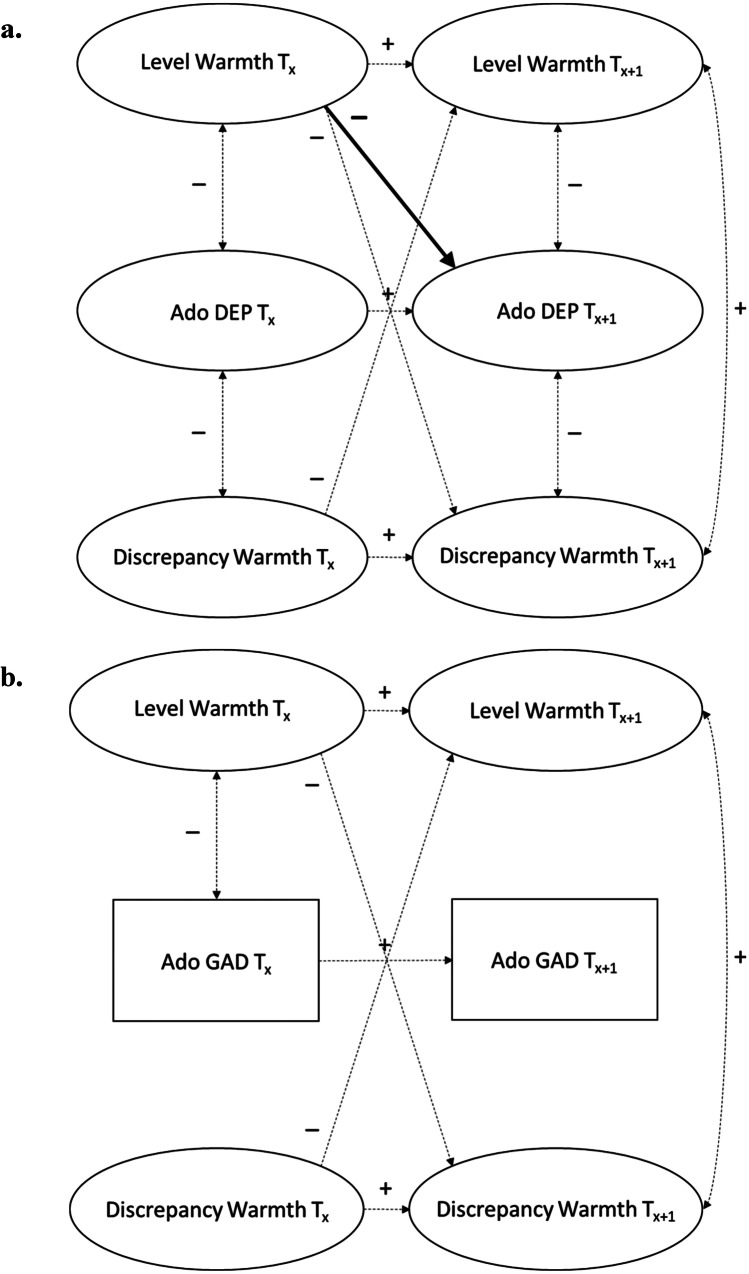
Simplified Visualization of the Main Findings of the Latent Congruence Models on the Association between Warmth in the Mother-Adolescent Relationship and **(a)** Adolescent Depressive Symptoms / **(b)** Adolescent GAD Symptoms (*N* = 497). *Note*. Only significant associations are visualized. The significant main findings are presented in bold lines, whereas the other significant associations are presented in dashed lines. *Ado* adolescent, *DEP* depressive symptoms, *GAD* Generalized Anxiety Disorder symptoms

The time-invariant baseline LCM showed acceptable to good fit to the data for the association between warmth in the mother-adolescent relationship and both adolescent depressive symptoms, χ^2^_SB_(2187) = 3490.58, CFI = 0.913, RMSEA [90% CI] = 0.035 [0.032, 0.037], SRMR = 0.092, and adolescent GAD symptoms, χ^2^_SB_(2187) = 3508.61, CFI = 0.913, RMSEA [90% CI] = 0.035 [0.033, 0.037], SRMR = 0.091. Stepwise freeing all bivariate within-time correlated changes, Δχ^2^_SB_(4) ≤ 6.11, *p*s ≥ 0.191 and Δχ^2^_SB_(4) ≤ 7.49, *p*s ≥ 0.112, and cross-lagged paths, Δχ^2^_SB_(4) ≤ 7.71, *p*s ≥ 0.103 and Δχ^2^_SB_(4) ≤ 6.98, *p*s ≥ 0.137, over time did not significantly improve model fit in neither the adolescent depressive nor GAD symptom model, respectively, with the exception of the correlated change between adolescent depressive symptoms and discrepancies between mothers and adolescents in their reports of warmth, Δχ^2^_SB_(4) = 12.55, *p*s = 0.014; χ^2^_SB_(2183) = 3477.73, CFI = 0.913, RMSEA [90% CI] = 0.035 [0.032, 0.037], SRMR = 0.092. For reasons of parsimony, all longitudinal parameters were therefore kept constrained to be equal over time in both models, except for the correlated change between adolescent depressive symptoms and discrepancies between mothers and adolescents in their reports of warmth. Results of the final LCMs are reported in Table 1 and a summary of the main findings is visualized in Fig. 2.

Most important to this study, in contrast to our hypothesis both adolescent depressive and GAD symptoms did not significantly predict levels of warmth 1-year later (*p* = 0.39 and *p* = 0.81, respectively). Higher levels of warmth did significantly predict lower adolescent depressive symptoms (βs = -0.04 – -0.05, *p* = 0.01), but not GAD symptoms (*p* = 0.56), 1-year later. These results suggest a negative unidirectional longitudinal association from levels of warmth to adolescent depressive symptoms over time, which is partially consistent with our hypothesis in line with interpersonal risk models. In addition, adolescent depressive and GAD symptoms did not significantly predict discrepancies between mothers and adolescents in their reports of warmth 1-year later (*p* = 0.20 and *p* = 0.36, respectively). Furthermore, in contrast to our hypothesis, discrepancies in reports of warmth did not significantly predict adolescent depressive or GAD symptoms 1-year later (*p* = 0.25 and *p* = 0.41, respectively). Thus, a slightly different pattern of findings was found for adolescent depressive symptoms and adolescent GAD symptoms concerning levels of warmth in the mother-adolescent relationship, although generally non-significant longitudinal associations were found for both internalizing symptoms.

### Sensitivity Analyses

Sensitivity analyses included adolescent gender as a time-invariant covariate of all variables in the final LCMs. Importantly, including gender as a time-invariant covariate in the analyses did not affect any of the conclusions concerning associations between adolescent depressive or GAD symptoms and both levels and discrepancies in mother-adolescent conflict as well as mother-adolescent warmth (please see Online Resource 3 for more details).

## Discussion

This 6-year longitudinal community study examined potential differential longitudinal associations among adolescent depressive and GAD symptoms and both levels of and discrepancies in adolescents’ and mothers’ perceptions of conflict and warmth in the mother-adolescent relationship (as reflections of negative and positive aspects of mother-adolescent relationship quality, respectively) to get to a more nuanced understanding of direction of effects in these associations over time. Results from Latent Congruence Models suggested that both higher depressive and higher GAD symptoms of adolescents significantly predicted higher levels of conflict in the mother-adolescent relationship 1-year later as well as larger discrepancies in adolescents’ and mothers’ perceptions of conflict 1-year later (with adolescents reporting higher levels of conflict than mothers). In turn, higher levels of conflict in the mother-adolescent relationship significantly predicted both higher depressive and higher GAD symptoms of adolescents 1-year later. These results thus suggest positive bidirectional longitudinal associations between levels of conflict in the mother-adolescent relationship and adolescent internalizing symptoms over time and a positive unidirectional longitudinal association from adolescent internalizing symptoms to discrepancies between mothers and adolescents in their reports of conflict. For levels of and discrepancies in adolescents’ and mothers’ perceptions of warmth in the mother-adolescent relationship, no significant longitudinal associations were found with either adolescent depressive or GAD symptoms over time, except for a negative unidirectional longitudinal association from levels of warmth to adolescent depressive symptoms. In sum, findings suggested robust support for transactional associations between higher levels of conflict and higher levels of both adolescent depressive symptoms and adolescent GAD symptoms over time.

### Mother-adolescent Relationship Quality and Adolescent Internalizing Symptoms

Findings concerning *levels* of mother-adolescent relationship quality suggested consistent bidirectional longitudinal associations between higher levels of both adolescent depressive and GAD symptoms and higher levels of conflict over time. These findings thereby support a *transactional model* (Rudolph, 2009; Sameroff, 2009) for both adolescent depressive and GAD symptoms, in which higher mother-adolescent conflict both predicts (in line with *interpersonal risk models*) and is predicted by (in line with *interpersonal scar models*) higher adolescent internalizing symptoms over time. Potential explaining mechanisms according to *interpersonal risk models* may involve interpersonal stress as well as maladaptive internal working models of the self and others caused by higher mother-adolescent conflict that may undermine effective emotion and stress regulation, coping, and interpersonal competence and may thereby increase the risk for adolescent internalizing symptoms (Bolton et al., 2009; for a review, see Rudolph et al., 2016). Furthermore, *interpersonal scar models* suggest that adolescents with higher levels of internalizing symptoms may in turn rely on negative interpersonal interaction styles that eventually induce a negative mood in their interaction partners (Coyne, 1976; Joiner & Coyne, 1999) or generate stress in their interpersonal relationships (Hammen, 2006) and may thereby (further) deteriorate the quality of the mother-adolescent relationship. Hence, this finding points to a vicious cycle over time in which the mother-adolescent relationship becomes increasingly negative, including higher levels of conflict, and adolescents experience increasing internalizing symptoms, through processes of relationship erosion, stress generation, and maladaptive working models. Importantly, higher levels of mother-adolescent conflict and higher levels of adolescent depression and GAD do not just appear to co-occur (i.e., a co-occurrence of problems), but appear to reinforce each other over time. Furthermore, whereas some of these processes have been proposed in the context of youth depression, these transactional associations appear to apply to adolescent GAD symptoms as well.

At the same time, findings concerning warmth in the mother-adolescent relationship merely suggested *unidirectional effects* over time with lower levels of warmth significantly predicting higher adolescent depressive symptoms, but not GAD symptoms, 1-year later (in line with *interpersonal risk models*; for a review, see Rudolph et al., 2016). Thus, this finding suggests differential longitudinal associations for adolescent depressive vs. GAD symptoms and warmth vs. conflict in the mother-adolescent relationship. Concerning the first, a more important role of parental acceptance has been suggested in youth depression compared to anxiety (McLeod et al., 2007a, b). This may be because processes of interpersonal stress and internalization of non-supportive parent–child interactions in negative schemas of the self and interpersonal relationships underlying this association may pose a stronger risk for adolescent depression (Bolton et al., 2009; Rudolph et al., 2016) than adolescent GAD. Concerning the latter, the general principle of “bad is stronger than good” (Baumeister et al., 2001) might explain why more longitudinal associations between conflict than warmth in the mother-adolescent adolescent internalizing symptoms were found. On the positive side, this unidirectional longitudinal association suggests that adolescent internalizing symptoms do not predict support erosion in the mother-adolescent relationship, but merely predict (further) increases in levels of conflict. This might suggest that warmth reflects a more stable attitude or working model within the mother-adolescent relationship that is not as easily affected by relationship or interaction difficulties resulting from adolescent internalizing symptoms as conflict. When adolescent internalizing problems make mother-adolescent interactions more difficult, this might result in daily hassles (i.e., increased conflict) but not necessarily directly decrease the stability of the mother-adolescent relationship and their affectional bond. Yet, we should emphasize that the overall pattern of findings was quite consistent across both adolescent depressive symptoms and adolescent GAD symptoms with respect to longitudinal associations with levels of mother-adolescent relationship quality.

### Mother-adolescent Discrepancies and Adolescent Internalizing Symptoms

Findings concerning *discrepancies* in adolescents’ and mothers’ perceptions of mother-adolescent relationship quality suggested consistent unidirectional longitudinal associations from higher levels of both adolescent depressive and GAD symptoms to higher levels of discrepancies in conflict, but not warmth, over time. Thereby, internalizing symptoms not only appeared to contribute to higher multi-informant levels of mother-adolescent conflict, but also to an increased gap between adolescents’ and mothers’ perceptions of their relationship quality. Specifically, in line with *interpersonal scar* or *symptom-driven* models, adolescent internalizing symptoms predicted increased adolescent-reported conflict compared to mother-reported conflict over time. According to the *Depression-Distortion Hypothesis* (Richters, 1992), adolescents’ ‘overreport’ of conflict relative to their mothers could be because higher levels of adolescent internalizing symptoms go together with certain cognitive (interpretation) biases or negative subjective experiences of social relationships causing adolescents to perceive more conflict in the mother-adolescent relationship than their mothers. Importantly, in this study no support for *Goodness of Fit* models (e.g., Lerner et al., 1986; Thomas & Chess, 1977), *Stage-Environment Fit Theory* (Eccles et al., 1993), or the Diverging Operations in the Operations Triad Model (De Los Reyes & Ohannessian, 2016) was found, as mother-adolescent discrepancies in both conflict and warmth did not significantly predict higher adolescent internalizing symptoms.

Thus, *levels* of mother-adolescent relationship quality appear to present a stronger risk or protective factor for adolescent depressive and GAD symptoms than *discrepancies* in perceptions of mother-adolescent relationship quality. As some empirical evidence exists for the negative impact of mother-adolescent discrepancies on adolescent functioning (e.g., Guion et al., 2009; Hou et al., 2018; Pelton & Forehand, 2001), future longitudinal research should continue to employ LCMs to examine the robustness of this finding in other samples and contexts over time while controlling for concurrent associations and the relative stability of these constructs over time as well as levels of conflict and warmth.

### Strengths, Limitations, and Directions for Future Research

This study has several strengths. First, it applied longitudinal Latent Congruence Models, in which both levels and discrepancies in adolescents’ and mothers’ perceptions of mother-adolescent relationship quality were analyzed in the same analytical model, in a relatively large community sample of adolescents followed from early to late adolescence. Second, it examined both warmth and conflict in the mother-adolescent relationship in association with both adolescent depressive symptoms and GAD symptoms. Thereby, the findings of this study contribute to a more nuanced understanding in terms of potential risk and protective factors as well as potential consequences of adolescent internalizing symptoms in the context of different aspects of the mother-adolescent relationship in a critical developmental period.

Yet, this study also has some limitations. First, our community sample was quite homogeneous, consisting largely of medium-to-high SES, two-parent Dutch families that were relatively well-adjusted in terms of relatively low levels of conflict, high levels of warmth, and low levels of adolescent internalizing symptoms. Also, this study used data collected more than a decade ago (from 2006 to 2012) as part of an ongoing intergenerational longitudinal community study. Future research with more diverse and present-day samples may want to examine the generalizability of our findings to different populations and conditions, including clinical populations. Different levels of, or larger variation in, the constructs of interest in more diverse samples may not necessarily affect associations between adolescent internalizing symptoms and levels of parental conflict or warmth–assuming linear associations– (in line with developmental psychopathology; Cicchetti & Rogosch, 2002; Rutter & Sroufe, 2000). However, future research is needed to examine how our findings on associations between adolescent internalizing symptoms and discrepancies in adolescents’ and parents’ perceptions of their relationship might be impacted by different sample characteristics.

Second, meta-analyses (McLeod et al., 2007a, b; Pinquart, 2017; Yap et al., 2014) have suggested that other (sub)dimensions of the parent-adolescent relationship, such as aspects of parental control, may also be important to consider in potential differential association with adolescent internalizing symptoms. For example, parental control has been more strongly associated with child and adolescent anxiety symptoms than depression (McLeod et al., 2007a, b); Excessive overinvolvement or low autonomy-granting or -support may prevent youth from experiencing control in age-appropriate contexts, limiting their development of self-efficacy and opportunities to learn appropriate (coping) skills, thereby increasing their sense of vulnerability to threat and particularly anxiety (Chorpita & Barlow, 1998). In addition, from a family systems perspective it would also be important to examine aspects of father-adolescent relationship quality, as suggestions for differential associations have been found in the context of parent-adolescent discrepancies and adolescent internalizing symptoms (e.g., Nelemans et al., 2016). Also, an interesting direction for future research would be to focus on parents and their adolescent as triadic unit, for example focusing on both parents as dyadic unit and potential parent-discrepancies in association with adolescent functioning (Stolz et al., 2005).

## Conclusion

In conclusion, this 6-year longitudinal community study provides a more nuanced understanding of the direction of effects among adolescent depressive and GAD symptoms and both levels of and discrepancies in adolescents’ and mothers’ perceptions of conflict and warmth in the mother-adolescent relationship. Specifically, findings suggested (1) that conflict in the mother-adolescent relationship was more strongly associated with both adolescent depressive and GAD symptoms over time than warmth; (2) support for both *interpersonal scar* or *symptom-driven* models (concerning both levels of and mother-adolescent discrepancies in conflict) and *interpersonal risk* models (concerning levels of both conflict and warmth); and (3) strong consistency in the pattern of findings across adolescent depressive and GAD symptoms, with comparable longitudinal associations for conflict in the mother-adolescent relationship and one longitudinal unidirectional association from warmth in the mother-adolescent relationship to adolescent depression specifically.

## Supplementary Information

Below is the link to the electronic supplementary material.

Supplementary file1 (PDF 163 KB)

Supplementary file2 (PDF 103 KB)

Supplementary file3 (PDF 111 KB)

## Data Availability

Data of the RADAR (Research on Adolescent Development And Relationships) study were used (https://doi.org/10.17026/dans-zrb-v5wp).

## Footnotes

1 Originally, this subscale consisted of eight items to assess features of warmth/support in the mother-adolescent relationship (Furman & Buhrmester, 1985). However, three items failed to fit with our research aim and were removed from our analyses, because they asked adolescents and mothers to report on different aspects of the mother-adolescent relationship. Specifically, these items did not ask both informants to report on the same person’s behavior or a characteristic of the relationship, but rather changed ‘directionality’ or ‘person of reference’. For example, adolescents were asked to report on “How much do you share your secrets and private feelings with your mother?” (i.e., adolescent mother), whereas mothers were asked to report on “How much do you share your secrets and private feelings with your child?” (i.e., mother adolescent, instead of adolescent mother). Therefore, these three items did not assess the same construct according to multiple informants–which is required for our Latent Congruence Modeling analyses, because of the measurement invariance tests, but also research on informant discrepancies in general–and were not included in our analyses.

2 The full correlation matrix is available from the first author upon request.
