# Supplementary material for: Do You See What I See? Longitudinal Associations Between Mothers’ and Adolescents’ Perceptions of Their Relationship and Adolescent Internalizing Symptoms
Source: Res Child Adolesc Psychopathol. 2022 Sep 17;51(2):177–92. doi: 10.1007/s10802-022-00975-5 (PMC9867686; doi:10.1007/s10802-022-00975-5)
Supplement: Supplementary file 1 — Supplementary file1 (PDF 163 KB) [file 10802_2022_975_MOESM1_ESM.pdf]

Online Resource 1

Article title: Do You See What I See? Longitudinal Associations Between Mothers' and Adolescents' Perceptions of Their Relationship and Adolescent Internalizing Symptoms

Journal name: *Research on Child and Adolescent Psychopathology*

Author names: Stefanie A. Nelemans, Stefanos Mastrotheodoros, Leyla Çiftçi, Wim Meeus & Susan Branje

Affiliation and e-mail address of the corresponding author: Stefanie A. Nelemans; Department of Youth and Family, Utrecht University, Utrecht, the Netherlands; s.a.nelemans@uu.nl

Method

Statistical Analyses

Measurement invariance analyses. Before running our main analyses, we conducted two crucial tests for measurement invariance concerning conflict and warmth in the parent-adolescent relationship. We first tested for *longitudinal measurement invariance within informants* to ensure that the assessment of conflict and warmth in the mother-adolescent relationship did not change over time for both adolescents and mothers. Building on these final longitudinal measurement invariance models, we subsequently tested for *measurement invariance across informants* to make sure that the assessment of conflict and warmth in the mother-adolescent relationship did not differ between informants over time.

All tests for measurement invariance were conducted in M*plus* Version 8.3, using maximum likelihood estimation with standard errors and chi square robust to non-normality (i.e., MLR estimator; Muthén & Muthén, 1998-2017). Model fit was assessed with the Comparative Fit Index (CFI), with values ≥ .90 indicating acceptable fit and values ≥ .95 indicating good fit, the Root Mean Squared Error of Approximation (RMSEA) and its 90% confidence interval (90% CI), with values ≤ .08 indicating acceptable fit and values of ≤ .05 indicating good fit, and the Standardized Root Mean Square Residual (SRMR), with values ≤ .10 indicating acceptable fit and values of ≤ .05 indicating good fit (Hu & Bentler, 1999; Kline, 2005). Four consecutive and nested Confirmatory Factor Analyses (CFAs) were specified with increasing equality constraints and the change in model fit of each model to the next was examined. If specifying increasing constraints from one model to the next did not significantly worsen model fit according to at least two of the three fit indices, longitudinal or cross-informant measurement invariance was assumed to hold. Following recommendations by Chen (2007), we used these cutoff criteria for acceptable change in fit indices: ΔCFI ≤ -.010, ΔRMSEA ≤ .015, and ΔSRMR ≤ .030 for metric invariance, but

ΔSRMR ≤ .010 for scalar and strict invariance. The CFAs were parameterized by constraining the first item factor loading to 1 and intercept to 0, so the latent factor means and variances could be estimated. Following recommendations by Vandenberg and Lance (2000), all CFAs included residual covariances between the same items across successive years (e.g., between T_1 and T_2 and between T_2 and T_3).

As a first step, we tested for *configural invariance*. We specified a CFA in which all items loaded on a single warmth or conflict factor every wave, across time/informants, without specifying any equality constraints across time/informants. When this model yields adequate fit, configural invariance is generally assumed, suggesting that the same 1-factor structure and pattern of factor loadings holds across time/informants. In the second step, we tested for *metric* (or *weak factorial*) *invariance* by adding equality constraints to the factor loadings across time/informants, thereby testing whether the size of the factor loadings is equal across time/informants. When these constraints do not significantly worsen model fit, all items are considered equally important to the measurement of the warmth or conflict across time/informants. In the third step, we tested for *scalar* (or *strong factorial*) *invariance* by adding equality constraints to the item intercepts across time/informants. When these constraints do not significantly worsen model fit, the levels and scaling of the items are considered equal across time/informants. In the fourth and final step, we tested for *strict* (or *full uniqueness*) *invariance* by adding equality constraints to the residual variances of the items across time/informants. When these constraints do not significantly worsen model fit, the amount of measurement error of each item is considered the same across time/informants. If strict measurement invariance holds over time, this suggests that conflict and warmth in the mother-adolescent relationship are assessed identically across time/informants.

Results

Tests for Longitudinal Measurement Invariance of Mother-Adolescent Relationship Quality Across Informants

Conflict. For each informant separately, we started with a longitudinal CFA model with all 6 conflict-items loading on one latent factor for every wave without specifying any equality constraints over time to test for longitudinal configural invariance. Fit of the longitudinal configural model was acceptable-to-good for both mothers, $\chi^2_{SB}(573) = 1071.35$, RMSEA [90% CI] = .042 [.038, .046], CFI = .950, SRMR = .036, and adolescents, $\chi^2_{SB}(573) = 967.18$, RMSEA [90% CI] = .037 [.033, .041], CFI = .966, SRMR = .032. Therefore, we further successively tested metric, scalar, and strict longitudinal measurement invariance models for each informant separately. Table S1 shows the model fit statistics of all longitudinal measurement invariance models. All changes in CFI, RMSEA, and SRMR were below the cutoff criteria for both informants. This suggests that the increasing equality constraints over time specified in each subsequent model did not significantly worsen model fit. Thus, strict longitudinal measurement invariance held for the 6-item conflict subscale of the NRI for both informants from early to late adolescence. In other words, conflict in the mother-adolescent relationship was assessed similarly across adolescence for each informant separately.

Subsequently, we tested for different levels of measurement invariance across the informants to assess whether the 6-item conflict subscale of the NRI was measured similarly over time for mothers and adolescents. We therefore started with the configural invariance model, which consisted of a combined longitudinal CFA model for both informants including all longitudinal equality constraints associated with the established strict longitudinal measurement invariance for each informant but did not include any equality constraints *between* mothers and adolescents. Fit of the configural model was acceptable-to-good, $\chi^2_{SB}(2566) = 3864.56$, RMSEA [90% CI] = .032 [.030, .034], CFI = .946, SRMR = .040.

Therefore, we further successively tested metric, scalar, and strict measurement invariance models across the two informants. Table S2 shows the model fit statistics of all measurement invariance models. All changes in CFI, RMSEA, and SRMR were below the cutoff criteria. This suggests that the increasing equality constraints across the two informants specified in each subsequent model did not significantly worsen model fit. Thus, strict longitudinal measurement invariance held for the 6-item conflict subscale of the NRI across both informants from early to late adolescence. In other words, conflict in the mother-adolescent relationship was assessed similarly across adolescence across both informants.

Warmth. For each informant separately, we started with a longitudinal CFA model with all 5 warmth-items loading on one latent factor every wave without specifying any equality constraints over time to test for longitudinal configural invariance. Fit of the longitudinal configural model was acceptable-to-good for both mothers, $\chi^2_{SB}(365) = 532.79$, RMSEA [90% CI] = .030 [.025, .036], CFI = .972, SRMR = .052, and adolescents, $\chi^2_{SB}(365) = 642.89$, RMSEA [90% CI] = .039 [.034, .044], CFI = .951, SRMR = .056. Therefore, further successively tested metric, scalar, and strict longitudinal measurement invariance models for each informant separately. Table S1 shows the model fit statistics of all longitudinal measurement invariance models. All changes in CFI, RMSEA, and SRMR were below the cutoff criteria for at least two of the three fit indices for both informants. Thus, strict longitudinal measurement invariance held for the 5-item warmth subscale of the NRI for both informants from early to late adolescence. In other words, warmth in the mother-adolescent relationship was assessed similarly across adolescence for each informant separately.

Subsequently, we tested for different levels of measurement invariance across the informants to assess whether the 5-item warmth subscale of the NRI was measured similarly over time for mothers and adolescents. We therefore started with the configural invariance

model, which consisted of a combined longitudinal CFA model for both informants including all longitudinal equality constraints associated with the established strict longitudinal measurement invariance for each informant but did not include any equality constraints *between* mothers and adolescents. Fit of the configural model was acceptable-to-good, $\chi^2_{SB}(1724) = 2411.79$, RMSEA [90% CI] = .028 [.026, .031], CFI = .948, SRMR = .064. Therefore, we further successively tested metric, scalar, and strict measurement invariance models across the two informants. Table S2 shows the model fit statistics of all measurement invariance models. All changes in CFI, RMSEA, and SRMR were below the cutoff criteria for at least two of the three fit indices, except for the CFI and RMSEA in the scalar invariant model. Based on the modification indices, we therefore constructed a partial longitudinal scalar model by removing the across-informant constraint on item 14 (“How much do you care about your mother/child?”) and let the mean/intercept of item 14 be freely estimated between informants. All changes in CFI, RMSEA, and SRMR were then below the cutoff criteria for at least two of the three fit indices. This suggests that the increasing equality constraints across the two informants specified in each subsequent model did not significantly worsen model fit. Thus, strict longitudinal measurement invariance held for the 5-item warmth subscale of the NRI across both informants from early to late adolescence. In other words, warmth in the mother-adolescent relationship was assessed similarly across adolescence across both informants.

References

Chen, F. F. (2007). Sensitivity of goodness of fit indexes to lack of measurement invariance. *Structural Equation Modeling, 14*, 464-504. doi:10.1080/10705510701301834

Hu, L., & Bentler, P. M. (1999). Cutoff criteria for fit indexes in covariance structure analysis: Conventional criteria versus new alternatives. *Structural Equation Modeling, 6*, 1-55. doi:10.1080/10705519909540118

Kline, R. B. (2005). *Principles and practice of structural equation modeling* (2nd ed.). New York, NY: Guilford Press.

Muthén, L. K. & Muthén, B. O. (1998-2017). *Mplus user's guide (*8th ed.). Los Angeles, CA: Muthén & Muthén.

Vandenberg, R. J., & Lance, C. E. (2000). A review and synthesis of the measurement invariance literature: Suggestions, practices, and recommendations for organizational research. *Organizational Research Methods, 3*, 4-70. doi:10.1177/109442810031002

Table S1.1

Model Fit Statistics for the Different Levels of Longitudinal Measurement Invariance Across 6 Successive Years (N = 497)

Variable / Model	χ^2_{SB} (df)	CFI	ΔCFI	RMSEA [90% CI]	ΔRMSEA	SRMR	ΔSRMR
Adolescent-report warmth							
Model 1: Configural Invariance[a,b]	642.891 (365)	.951		.039 [.034, .044]		.056	
Model 2: Metric Invariance	675.226 (385)	.949	-.002	.039 [.034, .044]	.000	.066	.010
Model 3: Scalar Invariance	705.913 (405)	.947	-.002	.039 [.034, .043]	.000	.068	.002
Model 4: Strict Invariance	713.177 (430)	.950	.003	.036 [.032, .041]	-.003	.084	.016
Mother-report warmth							
Model 1: Configural Invariance[a,b]	532.789 (365)	.972		.030 [.025, .036]		.052	
Model 2: Metric Invariance	549.476 (385)	.973	.001	.029 [.024, .035]	-.001	.056	.004
Model 3: Scalar Invariance	598.103 (405)	.968	-.005	.031 [.026, .036]	.002	.057	.001
Model 4: Strict Invariance	602.085 (430)	.972	.004	.028 [.023, .034]	-.003	.059	.002
Adolescent-report conflict							
Model 1: Configural Invariance[a]	967.18 (573)	.966		.037 [.033, .041]		.032	

Model 2: Metric Invariance	996.42 (598)	.965	-.001	.037 [.033, .041]	.000	.035	.003
Model 3: Scalar Invariance	1088.07 (623)	.960	-.005	.039 [.035, .043]	-.002	.036	.001
Model 4: Strict Invariance	1140.06 (653)	.958	-.002	.039 [.035, .042]	.000	.040	.004
Mother-report conflict							
Model 1: Configural Invariance[a]	1071.349 (573)	.950		.042 [.038, .046]		.036	
Model 2: Metric Invariance	1111.431 (598)	.949	-.001	.042 [.038, .045]	.000	.040	.004
Model 3: Scalar Invariance	1178.295 (623)	.945	-.004	.042 [.039, .046]	.000	.039	-.001
Model 4: Strict Invariance	1194.112 (653)	.946	.001	.041 [.037, .044]	-.001	.044	.005

Note. All SBχ^2 values were significant at $p < .001$.

[a]For reasons of parsimony, the configural measurement invariance models included time-invariant residual covariances between the same items over successive years, which did not significantly worsen model fit in terms of changes in the model fit indices compared to the configural measurement invariance models including time-variant residual covariances between the same items over successive years.

[b]The model fit of the initial configural measurement invariance models indicated unacceptable model fit for mothers, CFI < .90, and just-acceptable fit for adolescents, CFI = .90. Although the other model fit indices indicated (just-)acceptable fit, we aimed to improve the CFI. Modification indices suggested that the model fit strongly improved by including residual covariances between the same items across all possible years, instead of just subsequent years, for both informants. We therefore included these additional residual covariances in our models

for both informants.

Table S1.2

Model Fit Statistics for the Different Levels of Longitudinal Measurement Invariance Across Informants (N = 497)

Variable / Model	χ^2_{SB} (df)	CFI	ΔCFI	RMSEA [90% CI]	ΔRMSEA	SRMR	ΔSRMR
Warmth							
Model 1: Configural Invariance[a]	2411.79	.948		.028 [.026, .031]		.064	
Model 2: Metric Invariance	2482.82	.943	-.005	.030 [.027, .032]	.002	.073	.009
Model 3: Scalar Invariance	2788.35	.921	-.022	.035 [.033, .037]	.005	.090	.017
Model 3: Partial Scalar Invariance[b]	2497.49	.942	-.001	.030 [.027, .032]	.000	.074	.001
Model 4: Strict Invariance	2553.07	.939	-.003	.031 [.028, .033]	.001	.085	.011
**Model 5: Partial Strict Invariance+[c]**	**2687.44**	**.930**	**-.009**	**.033 [.030, .035]**	**.002**	**.085**	**.000**
Conflict							
Model 1: Configural Invariance[a]	3864.56	.946		.032 [.030, .034]		.040	
Model 2: Metric Invariance	3952.40	.943	.003	.033 [.031, .035]	.001	.043	.003
Model 3: Scalar Invariance	4060.30	.939	.004	.034 [.032, .036]	.001	.042	.001
Model 4: Strict Invariance	4226.39	.932	.007	.036 [.034, .038]	.002	.045	.003
**Model 5: Strict Invariance+[c]**	**4283.51**	**.930**	**.002**	**.036 [.034, .038]**	**.000**	**.045**	**.000**

Note. All SBχ^2 values were significant at $p < .001$. The final longitudinal measurement invariance model across informants used to build the Latent Congruence Models is in bold.

[a]Within each informant, equality constraints of the strict longitudinal invariance model were applied including time-invariant residual covariances between the same items across all years for warmth or across successive years for conflict.

[b]In the partial scalar invariance model, based on the modification indices we freely estimated the mean/intercept of item 14 (“How much do you care about your mother/child?”) between informants.

[c]For reasons of parsimony, we additionally tested whether the time-invariant residual covariances between the same items over successive years for each informant could also be constrained to be equal between the informants.
