# Supplementary material for: Do You See What I See? Longitudinal Associations Between Mothers’ and Adolescents’ Perceptions of Their Relationship and Adolescent Internalizing Symptoms
Source: Res Child Adolesc Psychopathol. 2022 Sep 17;51(2):177–92. doi: 10.1007/s10802-022-00975-5 (PMC9867686; doi:10.1007/s10802-022-00975-5)
Supplement: Supplementary file 2 — Supplementary file2 (PDF 103 KB) [file 10802_2022_975_MOESM2_ESM.pdf]

Online Resource 2

Article title: Do You See What I See? Longitudinal Associations Between Mothers' and Adolescents' Perceptions of Their Relationship and Adolescent Internalizing Symptoms

Journal name: *Research on Child and Adolescent Psychopathology*

Author names: Stefanie A. Nelemans, Stefanos Mastrotheodoros, Leyla Çiftçi, Wim Meeus & Susan Branje

Affiliation and e-mail address of the corresponding author: Stefanie A. Nelemans; Department of Youth and Family, Utrecht University, Utrecht, the Netherlands; s.a.nelemans@uu.nl

Figure 2.1

Simplified Graphical Representation of the Higher-Order Factor-Analytical Part of the Latent Congruence Model, Distinguishing Between Mean-Levels of Conflict or Warmth in the Mother-Adolescent Relationship and Discrepancies Between Mothers and Adolescents in their Reports of Conflict or Warmth

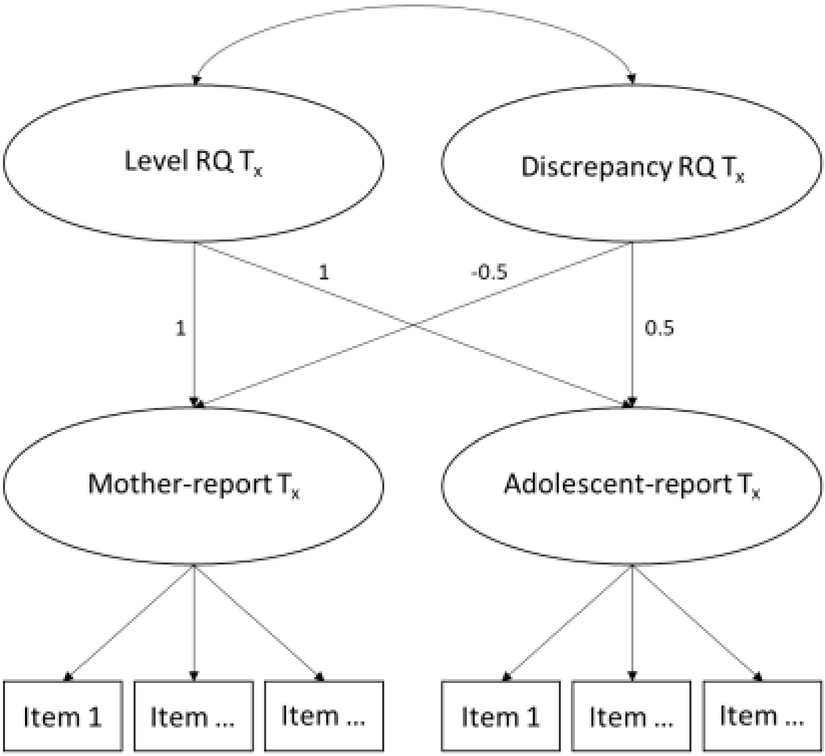

Note. RQ = mother-adolescent relationship quality (referring to either conflict or warmth in the mother-adolescent relationship)
