# Supplementary material for: Do You See What I See? Longitudinal Associations Between Mothers’ and Adolescents’ Perceptions of Their Relationship and Adolescent Internalizing Symptoms
Source: Res Child Adolesc Psychopathol. 2022 Sep 17;51(2):177–92. doi: 10.1007/s10802-022-00975-5 (PMC9867686; doi:10.1007/s10802-022-00975-5)
Supplement: Supplementary file 3 — Supplementary file3 (PDF 111 KB) [file 10802_2022_975_MOESM3_ESM.pdf]

Online Resource 3

Article title: Do You See What I See? Longitudinal Associations Between Mothers' and Adolescents' Perceptions of Their Relationship and Adolescent Internalizing Symptoms

Journal name: *Research on Child and Adolescent Psychopathology*

Author names: Stefanie A. Nelemans, Stefanos Mastrotheodoros, Leyla Çiftçi, Wim Meeus & Susan Branje

Affiliation and e-mail address of the corresponding author: Stefanie A. Nelemans; Department of Youth and Family, Utrecht University, Utrecht, the Netherlands; s.a.nelemans@uu.nl

Results

Descriptive Statistics

Table S3.1

Means and Standard Deviations of all Study Variables as well as Tests of Congruency Between Mother- and Adolescent-Reports of Conflict and Warmth in the Mother-Adolescent Relationship

	Adolescent report		Mother report		ΔM		r
Variable	*M*	*SD*	*M*	*SD*	*M*	*t*	
T_1 Ado DEP symptoms	1.63	0.49					
T_2 Ado DEP symptoms	1.50	0.50					
T_3 Ado DEP symptoms	1.53	0.52					
T_4 Ado DEP symptoms	1.56	0.54					
T_5 Ado DEP symptoms	1.54	0.51					
T_6 Ado DEP symptoms	1.59	0.55					
T_1 Ado GAD symptoms	1.39	0.39					
T_2 Ado GAD symptoms	1.32	0.38					

T_3 Ado GAD symptoms	1.36	0.43					
T_4 Ado GAD symptoms	1.39	0.44					
T_5 Ado GAD symptoms	1.37	0.44					
T_6 Ado GAD symptoms	1.42	0.47					
T_1 Conflict	1.66	0.58	1.52	0.53	0.14	5.33^{***}	$.45^{***}$
T_2 Conflict	1.71	0.67	1.55	0.54	0.16	5.93^{***}	$.54^{***}$
T_3 Conflict	1.75	0.67	1.52	0.50	0.22	7.87^{***}	$.50^{***}$
T_4 Conflict	1.79	0.69	1.55	0.56	0.25	7.86^{***}	$.48^{***}$
T_5 Conflict	1.80	0.72	1.50	0.54	0.31	9.60^{***}	$.49^{***}$
T_6 Conflict	1.74	0.65	1.48	0.54	0.26	9.15^{***}	$.54^{***}$
T_1 Warmth	4.39	0.55	3.96	0.51	0.43	14.22^{***}	$.21^{***}$
T_2 Warmth	4.30	0.61	3.91	0.50	0.39	13.00^{***}	$.33^{***}$
T_3 Warmth	4.22	0.66	3.95	0.50	0.26	7.81^{***}	$.26^{***}$
T_4 Warmth	4.14	0.68	3.94	0.53	0.20	6.04^{***}	$.34^{***}$
T_5 Warmth	4.17	0.69	3.92	0.53	0.24	7.00^{***}	$.37^{***}$
T_6 Warmth	4.13	0.73	3.96	0.55	0.16	4.53^{***}	$.42^{***}$

Note. Ado = Adolescent; DEP = depressive; GAD = Generalized Anxiety Disorder. All scores reflect averages across items, in which adolescent depressive symptoms could range from 1 to 4, adolescent GAD symptoms from 1 to 3, and both warmth and conflict in the mother-adolescent relationship from 1 to 5.

*** $p < .001$.

Table S3.2

Summary of Concurrent Correlations Between Adolescent- and Mother-Reports on Conflict and Warmth in the Mother-adolescent Relationship and Adolescent Depressive and GAD Symptoms Across Time

Variable	Ado DEP symptoms T_1-T_6	Ado GAD symptoms T_1-T_6
Ado conflict T_1-T_6	.29** – .37**	.19** – .27**
Ado warmth T_1-T_6	-.21** – -.31**	-.06 – -.16**
Mother-report conflict T_1-T_6	.23** – .30**	.11* – .20**
Mother-report warmth T_1-T_6	-.09 – -.16**	-.09 – .02

Note. Ado = Adolescent-report; DEP = depressive; GAD = Generalized Anxiety Disorder.

* $p < .05$. ** $p < .01$.

Sensitivity Analyses

Sensitivity analyses included adolescent gender as a time-invariant covariate of levels of mother-adolescent conflict or warmth, discrepancy in reports of mother-adolescent conflict or warmth, and adolescent depressive or GAD symptoms in the final LCMs. These models showed acceptable to good fit to the data for both adolescent depressive symptoms, $\chi^2_{SB}(3161) = 5261.15$, CFI = .920, RMSEA [90% CI] = .037 [.035, .038], SRMR = .085 and $\chi^2_{SB}(2246) = 3558.52$, CFI = .913, RMSEA [90% CI] = .034 [.032, .036], SRMR = .091, for conflict and warmth respectively, and adolescent GAD symptoms, $\chi^2_{SB}(3161) = 5307.55$, CFI = .919, RMSEA [90% CI] = .037 [.035, .039], SRMR = .084 and $\chi^2_{SB}(2250) = 3579.87$, CFI = .913, RMSEA [90% CI] = .034 [.032, .037], SRMR = .090, for conflict and warmth respectively.

Girls reported significantly higher levels of adolescent depressive symptoms, βs = .21 – .25, $p < .001$, and GAD symptoms, βs = .22 – .26, $p < .001$, than boys. Furthermore, mother-daughter dyads reported higher levels of warmth in their relationship, βs = .08 – .13, $p \leq .001$, and larger discrepancies in levels of warmth in their relationship, in which adolescents reported higher levels of warmth in comparison to their mothers, βs = .07 – .08, $p = .032 – .049$, than mother-son dyads. There were no significant gender differences in levels of mother-adolescent conflict, $p = .731 – .736$, or discrepancies in mother-adolescent conflict, $p = .105 – .124$. Importantly, including gender as a time-invariant covariate in the analyses did not affect any of the conclusions concerning associations between adolescent depressive or GAD symptoms and both levels and discrepancies in mother-adolescent conflict as well as mother-adolescent warmth.
